# Antibacterial, Antifungal, and Antioxidant Activities of Silver Nanoparticles Biosynthesized from *Bauhinia tomentosa* Linn

**DOI:** 10.3390/antiox10121959

**Published:** 2021-12-07

**Authors:** Senthil Renganathan, Sugunakala Subramaniyan, Nivetha Karunanithi, Preethi Vasanthakumar, Arne Kutzner, Pok-Son Kim, Klaus Heese

**Affiliations:** 1 Department of Bioinformatics, Marudupandiyar College, Thanjavur 613-403, India; rsenthil@mpi.edu.in; 2 Department of Bioinformatics, A.V.C. College, Mayiladuthurai 609-305, India; sugunakala@avccollege.net; 3 Department of Biotechnology, A.V.C. College, Mayiladuthurai 609-305, India; nivetha2161998@gmail.com; 4 Department of Biotechnology, Bharath College of Science and Management, Thanjavur 613-005, India; preethibiotech2009@gmail.com; 5 Department of Information Systems, College of Engineering, Hanyang University, 222 Wangsimni-ro, Seoul 133-791, Korea; kutzner@hanyang.ac.kr; 6 Department of Mathematics, Kookmin University, 77 Jeongneung-ro, Seoul 136-702, Korea; pskim@kookmin.ac.kr; 7 Graduate School of Biomedical Science and Engineering, Hanyang University, 222 Wangsimni-ro, Seoul 133-791, Korea

**Keywords:** antioxidant, bauhinia tomentosa, free radicals, microbial, nanoparticle, reactive oxygen species (ROS), silver

## Abstract

The biogenic synthesis of silver nanoparticles (AgNPs) has a wide range of applications in the pharmaceutical industry. Here, we synthesized AgNPs using the aqueous flower extract of *Bauhinia tomentosa* Linn. Formation of AgNPs was observed using ultraviolet-visible light spectrophotometry at different time intervals. Maximum absorption was observed after 4 h at 420 nm due to the reduction of Ag^+^ to Ag^0^. The stabilizing activity of functional groups was identified by Fourier-transform infrared spectroscopy. Size and surface morphology were also analyzed using scanning electron microscopy. The present study revealed the AgNPs were spherical in form with a diameter of 32 nm. The face-centered cubic structure of AgNPs was indexed using X-ray powder diffraction with peaks at 2θ = 37°, 49°, 63°, and 76° (corresponding to the planes of silver 111, 200, 220, 311), respectively. Energy-dispersive X-ray spectroscopy revealed that pure reduced silver (Ag^0^) was the major constituent (59.08%). Antimicrobial analyses showed that the biosynthesized AgNPs possess increased antibacterial activity (against *Staphylococcus aureus* (Gram-positive) and *Escherichia coli* (Gram-negative), with larger zone formation against *S. aureus* (9.25 mm) compared with that of *E. coli* (6.75 mm)) and antifungal activity (against *Aspergillus flavus* and *Candida albican* (with superior inhibition against *A. flavus* (zone of inhibition: 7 mm) compared with *C. albicans* (zone of inhibition: 5.75 mm)). Inhibition of 2,2-diphenyl-1-picrylhydrazyl (DPPH) radical scavenging activity was found to be dose-dependent with half-maximal inhibitory concentration (IC_50_) values of 56.77 μg/mL and 43.03 μg/mL for AgNPs and ascorbic acid (control), respectively, thus confirming that silver nanoparticles have greater antioxidant activity than ascorbic acid. Molecular docking was used to determine the mode of antimicrobial interaction of our biosynthesized *B. tomentosa* Linn flower-powder extract-derived AgNPs. The biogenic AgNPs preferred hydrophobic contacts to inhibit bacterial and fungal sustainability with reducing antioxidant properties, suggesting that biogenic AgNPs can serve as effective medicinal agents.

## 1. Introduction

Resistance to antibiotics and a wide variety of microorganisms in the public health system has become a major obstacle, and almost every single variant of microorganisms has developed antibiotic resistance [1,2]. According to contemporary ideas, nanoparticles, such as silver nanoparticles (AgNPs), can inhibit the growth of microbes [3,4,5]. Nanoparticles are structures with dimensions ranging from approximately 1 to 100 nm that exhibit significantly different physical (mechanical, optical, electrical) and chemical properties when compared with their larger counterparts [6,7]. Over the past 10 to 20 years, metal nanoparticles, and AgNPs in particular, have attracted attention due to their versatility and broad range of industrial and biomedical applications [8,9,10,11]. Potential uses include antimicrobial (antibacterial, antifungal, and antiviral) agents [12,13,14,15,16,17], biomedical device coatings, drug-delivery carriers, and imaging probes for diagnostic and optoelectronic applications [18,19,20,21,22]. AgNPs could mediate the antimicrobial activity by producing reactive oxygen species and free radicals causing cell wall damage, lipid peroxidation, protein denaturation, and nucleic acid and proton pump damage [4,23]. The use of biological methods and natural resources to synthesize AgNPs has increased considerably due to improved feasibility and high biocompatibility [22,24,25,26]. Biological synthetic pathways based on microorganisms or plant extracts have been widely explored for the production of AgNPs in several applications as they are environmentally friendly and often inexpensive. Moreover, plant-based extract-mediated AgNPs synthesis is more advantageous than other biological processes because it does not require stringent aseptic environments and strict monitoring of cell culture conditions [27,28,29,30,31,32,33,34]. The genus Bauhinia is a member of the Leguminosae family (subfamily Caesalpiniaceae) and consists of about 300 species. *Bauhinia tomentosa* is a South Indian shrub that has been applied in ayurvedic medicine for centuries based on its multiple beneficial effects, including antioxidant, anti-inflammatory, antitumor, antimicrobial, antiamoebic, antidiabetic, and antirheumatic properties as well as functioning as an analgesic and hypocholesterolemic agent [35,36,37,38,39]. Additionally, its extracts contain a diverse set of metabolites that could be possibly used in the reduction of silver ions, as a capping and stabilizing agent in the synthesis of nanoparticles [35,40,41,42]. Biological synthetic methods can produce AgNPs that are frequently more stable and less toxic than nanoparticles obtained using conventional methods [7,34,41,43,44,45,46,47]. The surface of green synthesized AgNPs has strong bioactive antioxidant and antimicrobial activity [34].

For the study of the potential mechanism of AgNPs-mediated antimicrobial effects, we selected DNA gyrase, cytochrome P450, and dihydrofolate reductase as potential candidate target proteins. DNA gyrase is categorized as topoisomerase II, an ATP-dependent enzyme involved in DNA transcription, replication, and chromosome segregation in Gram-negative and Gram-positive bacteria. In eukaryotes, cytochrome P450 catalyzes a variety of reactions and is an important enzyme in fungal primary and secondary metabolism. The cytochrome P450 enzyme is required for sterol biosynthesis in eukaryotic cells and is also the primary target of clinical drugs used to treat fungal pathogens. In addition, dihydrofolate reductase is a member of the reductase enzyme family, which is found in all living organisms and is required for fungal cell growth and proliferation. Thus, DNA gyrase, cytochrome P450, and dihydrofolate reductase are considered major therapeutic targets in drug delivery and design [48,49,50,51].

Accordingly, in the current study we biosynthesized AgNPs using *B. tomentosa* Linn flower powder extract as a natural source. We validated their antimicrobial activity and evaluated possible mechanisms of action via molecular docking analysis using DNA gyrase, cytochrome P450, and dihydrofolate reductase, respectively.

## 2. Materials and Methods

### 2.1. Materials

All chemicals used in this research, including antibiotics such as chloramphenicol, fluconazole, and silver nitrate (AgNO_3_), were of analytical grade and were purchased from Sigma-Aldrich (St. Louis, MO, USA).

### 2.2. Origin of B. tomentosa Linn flowers

*B. tomentosa* Linn flowers were collected from Sirkazhi (Nagapattinam District, Tamil Nadu, India; 11.2420° N, 79.7287° E), in December 2019. (An authenticated voucher specimen, No. 374, was deposited in the herbarium of the Department of Botany, Annamalai University, Chidambaram, Tamil Nadu, India). Plant material was washed with normal and distilled water, dried in the dark at room temperature, and ground to a fine powder, as described previously [52,53,54]. Ground *B. tomentosa* Linn flower powder (20 g) was soaked in distilled water for 24 h with mild shaking at room temperature, boiled for 10 min, filtered using Whatman grade 1 filter paper (Sigma-Aldrich), and concentrated by a rotary vacuum evaporator at 20 °C (EQUITRON, rotatory vacuum evaporator, Medica Instrument MFG. Co, Chennai, Tamil Nadu, India) to 1 mg/mL. Concentrated *B. tomentosa* Linn flower powder extract was stored at 4 °C until further use [54].

### 2.3. Preliminary Phytochemical Analysis

Qualitative phytochemical characterization of *B. tomentosa* Linn flower powder extracts (using 70% to 100% alcohol (methanol, ethanol), distilled water, or petroleum ether) followed established protocols described by Harborne [55] to identify and characterize the phytochemical constituents (including anthraquinones, coumarins, polyphenol, terpenoids, saponins, tannins, steroids, alkaloids, flavonoids, glycosides, triterpenoids, and terpenoids), as described previously [54,55].

### 2.4. Bacterial and Fungal Cultures

All bacterial and fungal cells were obtained from the Microbial Type Culture Collection and Gene Bank (MTCC) at the Institute of Microbial Technology, Chandigarh 160036, India. Species used included *Escherichia coli* (MTCC 732), *Staphylococcus aureus* (MTCC 3160), *Candida albicans* (MTCC 183), and *Aspergillus flavus* (MTCC 10180). The bacterial cultures were grown routinely in Luria Bertani broth and incubated at 37 °C (Technico Incubator, Model TLPPL 104, Technico Laboratory Products Pvt. Ltd., Chennai, Tamil Nadu, India). Fungal cultures were grown similarly on potato dextrose agar (PDA) and incubated at 27 °C for 7 days.

### 2.5. Synthesis of Silver Nanoparticles

A crude extract (5 mL) of *B. tomentosa* Linn flower powder was transferred into 45 mL of a 1 mM aqueous AgNO_3_ solution in an Erlenmeyer flask. The flask was incubated in the dark at room temperature for 5 h to minimize photoactivation of silver nitrate. The AgNP solution was purified by repeated centrifugation at 10,000 rpm for 15 min (REMI-C-30BL, Centrifuge, REMI Electrotecnik Limited, Chennai, Tamil Nadu, India) followed by washing of the pellets with deionized water and finally drying to collect the AgNPs [56,57,58].

### 2.6. Characterization of Silver Nanoparticles

The confirmation of biosynthesized *B. tomentosa* Linn flower powder-extract-derived AgNPs was accomplished using ultraviolet-visible light (UV-vis) spectrophotometry (Lambda 265, Perkin Elmer Health Sciences Pvt. Ltd., Chennai, Tamil Nadu, India; range: 300–800 nm) [58]. Characterization of AgNPs through Fourier-transform infrared spectroscopy (FTIR, Perkin Elmer FTIR-Spectrometer 1725 X, Perkin Elmer Health Sciences Pvt. Ltd., Chennai, Tamil Nadu, India) was used to detect the characteristic peaks of the functional groups attached to the surface of AgNPs in a spectral range of 400 to 4000 cm^−1^ [59,60]. Scanning electron microscopy (SEM) was used to study morphological information on the sample at the submicron scale and elemental information at the micron scale [61,62]. The dried samples were coated with gold (Polaron Emitech SC7640 sputter coater, Quorum Technologies Ltd., Newhaven, East Sussex, UK), and microscopic images were taken at 250× and a voltage of 10 kV by a Jeol JSM-6480LV SEM machine (JEOL Ltd., Tokyo, Japan) to characterize the particle size and morphology of the AgNPs. Energy-dispersive X-ray (EDX) analysis helped determine the elemental composition of the AgNPs [63]. X-ray powder diffraction (XRD) was applied for phase identification of the Cu Kα radiation (1.5405 Å) of the AgNPs (Philips PANanalytical X’Pert XRD System (model # 3040), Amsterdam, The Netherlands) [57,64]. 

### 2.7. Antimicrobial Activities of Biosynthesized AgNPs

#### 2.7.1. Antibacterial Activity 

The antibacterial activity of biosynthesized *B. tomentosa* Linn flower-powder extract-derived AgNPs was investigated against Gram-negative (*E. coli*) and Gram-positive (*S. aureus*) bacterial pathogens using agar disk diffusion [28,56,61,65,66]. Briefly, a nutrient agar medium was prepared in a Petri dish and the bacterial cultures were swabbed on test media with a sterile cotton swab. The discs were dipped with the following four components (30 μL): (i) biosynthesized AgNPs, (ii) *B. tomentosa* Linn flower powder extract, (iii) AgNO_3_ solution, and (iv) standard antibiotic solutions (chloramphenicol, 30 µg/mL). The dried discs were pressed gently over the surface of the culture-swabbed medium at equal distances to avoid overlapping of the inhibition zones. The plates were then incubated at 37 °C for 24 h. After incubation, the antibacterial activity of the biosynthesized AgNPs was evaluated according to the diameters of the clear inhibition zones [67].

#### 2.7.2. Antifungal Activity

Antifungal activity of biosynthesized *B. tomentosa* Linn flower-powder extract-derived AgNPs was analyzed against *A. flavus* and *C. albicans* by disk diffusion. The following four different components (30 μL) were applied on separate Whatman No. 1 filter paper discs 6 mm in diameter: (i) biosynthesized AgNPs, (ii) *B. tomentosa* Linn. flower powder extract, (iii) AgNO_3_ solution, and (iv) standard antifungal solution (fluconazole, 30 µg/mL). Each was allowed to dry before being placed on a PDA medium carrying the fungal strains and then incubated for 48 h. The diameter of the zones was measured in centimeters with the help of a scale, and the results were tabulated [28,58,68,69].

### 2.8. In Vitro Determination of Antioxidant Activity 

For antioxidant activity testing, every 1 mL of different concentrations (20, 40, 60, and 80 µg/mL) of biosynthesized *B. tomentosa* Linn flower-powder extract-derived AgNPs was mixed with 2 mL of freshly prepared 2,2-diphenyl-1-picrylhydrazyl solution (DPPH, 1 mM in methanol) and mixed meticulously. After the solution was incubated at room temperature, the absorbance of the solution was recorded at 517 nm using a UV-vis spectrophotometer (Lambda 265, Perkin Elmer). The free-radical scavenging activity was calculated as: [(absorbance at blank) − (absorbance at test)/(absorbance at blank)] × 100 [66].

### 2.9. Molecular Docking of Silver Nanoparticles

The structures of target proteins and small molecules (AgNPs, chloramphenicol, and fluconazole) were retrieved from the Protein Data Bank (PDB) and the PubChem database, respectively (PDB IDs: 3G7B [DNA gyrase, *S. aureus*], 4WUB [DNA gyrase, *E. coli*], 5TZI [cytochrome P450, *C. albicans*], and 6DRS [dihydrofolate reductase, *A. flavus*]). Molecular docking of AgNPs with receptors was accomplished through a Patch dock server (http://bioinfo3d.cs.tau.ac.il/PatchDock, accessed on 25 October 2021). The root-mean-square deviation was set at 4 Å, and receptor-ligand molecules were used for docking. Based on the scoring and interaction information, the top-ranked complexes were chosen for interaction studies and finding residues [54,70,71,72,73,74].

### 2.10. Statistical Analysis

Experiments were performed in at least three biological replicates (antibacterial, antifungal, and antioxidant assays) and data are presented as mean ± standard deviation. A Student’s t test was applied using SPSS software (IBM SPSS Statistics; Armonk, NY, USA) [75].

## 3. Results

### 3.1. Phytochemical Analysis

Qualitative phytochemical screening analysis of *B. tomentosa* Linn flower-powder extracts identified the phytochemical constituents in the alcohol and aqueous extracts. Aqueous extracts contained alkaloids, anthraquinone, coumarins, flavonoids, glycosides, polyphenol saponins, steroids, tannin, terpenoids, and triterpenoids. Alcoholic extracts did not obtain tannin (Table 1).

### 3.2. Biosynthesis of AgNPs

**Figure 1:**
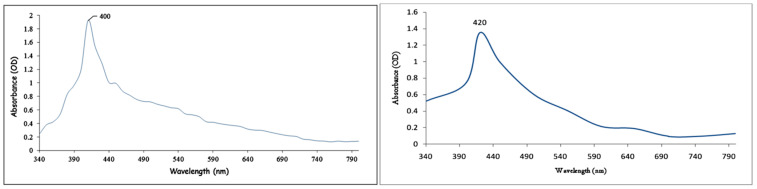
UV-vis spectra of biosynthesized *Bauhinia tomentosa* Linn flower-powder extract-derived AgNPs 0 h (**left**) and 4 h (**right**) of incubation of *B. tomentosa* Linn flower powder extract with AgNO_3_. Over a period of 4 h the absorption peak shifted from approximately 400 nm to 420 nm due to the reduction of Ag^+^ to Ag^0^, indicating that AgNPs were obtained.

Biosynthesis of *B. tomentosa* Linn flower-powder extract–derived AgNPs was monitored via the redox reaction (reduction of silver ions to metal and the formation of AgNPs) as recorded by UV-vis spectrophotometry (Figure 1). Over a period of 4 h the absorption peak shifted from approximately 400 nm to 420 nm due to the reduction of Ag^+^ to Ag^0^ (color shift from brown to yellowish), indicating that AgNPs were obtained.

### 3.3. Fourier-Transform Infrared Analysis of Biosynthesized AgNPs

**Figure 2:**
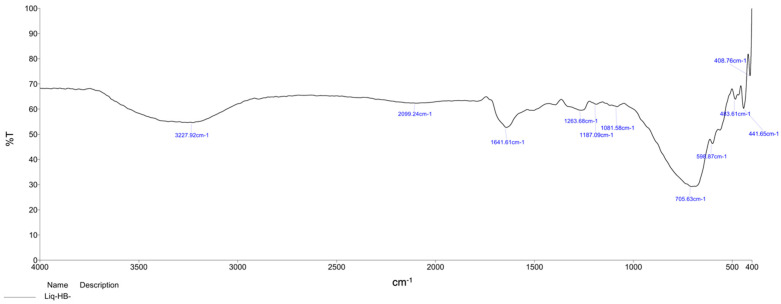
FTIR spectrum of biosynthesized *Bauhinia tomentosa* Linn flower-powder extract-derived AgNPs. Characterisitc peaks indicated the presence of aliphatic primary amine (N-H bonds, peak at 3227.92 cm^−1^), terminal alkyne (C=C bonds, peak at 2099.24 cm^−1^), imine/oxime (C=N bonds, peak at 1263.68 cm^−1^), ether (C-O bond, peak at 1187.09 cm^−1^) and aliphatic bromo components (C-Br bond, peak at 1081.58 cm^−1^) and also indicate the formation of reduced silver atoms (Ag^0^, peaks at 706.63 cm^−1^ to 408.76 cm^−1^).

FTIR spectroscopy (in a range from 400 to 4000 cm^−1^) was used to detect functional groups in biosynthesized *B. tomentosa* Linn flower-powder extract-derived AgNPs. Characteristic absorption peaks corresponding to the functional groups of secondary metabolites, such as aliphatic primary amine (N-H bonds, peak at 3227.92 cm^−1^), terminal alkyne (C=C bonds, peak at 2099.24 cm^−1^), imine/oxime (C=N bonds, peak at 1263.68 cm^−1^), ether (C-O bond, peak at 1187.09 cm^−1^) and aliphatic bromo components (C-Br bond, peak at 1081.58 cm^−1^), were evident. Formation of reduced silver atoms (Ag^0^, peaks at 706.63 cm^−1^ to 408.76 cm^−1^) and capping of the synthesized AgNPs by the phytochemicals present in the extract were also observed (Figure 2).

### 3.4. Energy-Dispersive Spectroscopy Analysis of Biosynthesized AgNPs

**Figure 3:**
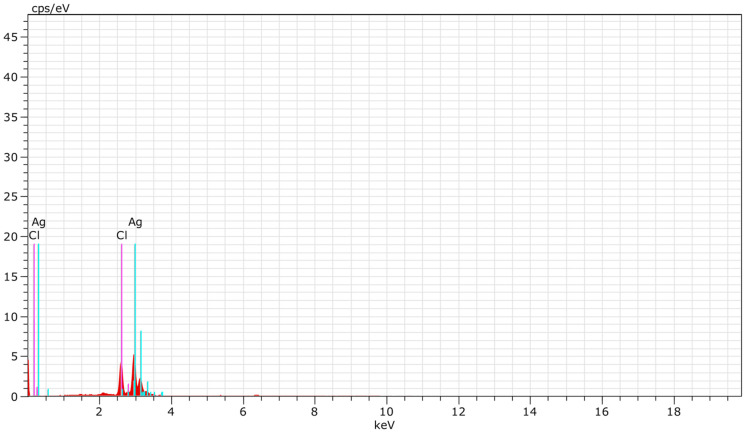
EDX spectroscopy spectrum of biosynthesized *Bauhinia tomentosa* Linn flower-powder extract-derived AgNPs. Signals for AgNPs appear at the expected position of 3 keV.

An EDX analysis of biosynthesized *B. tomentosa* Linn flower-powder extract-derived AgNPs revealed signal energy peaks for silver atoms in a range of 2–4 keV, with weaker signals for chloride; pure silver (59.08%) was the major element compared to chloride (41.92%) (Figure 3 and Table 2).

Strong signals of silver (59.08%) are clearly visible in the spectrum. The other signals can be attributed to the organic capping layer. The significant intensity of the peaks indicates the presence of a sufficient coating layer on the biosynthesized AgNPs [27,61,76].

The data indicate the successful biosynthesis of AgNPs with some amount of chlorine impurities [77].

### 3.5. X-ray Diffraction Analysis of Biosynthesized AgNPs

**Figure 4:**
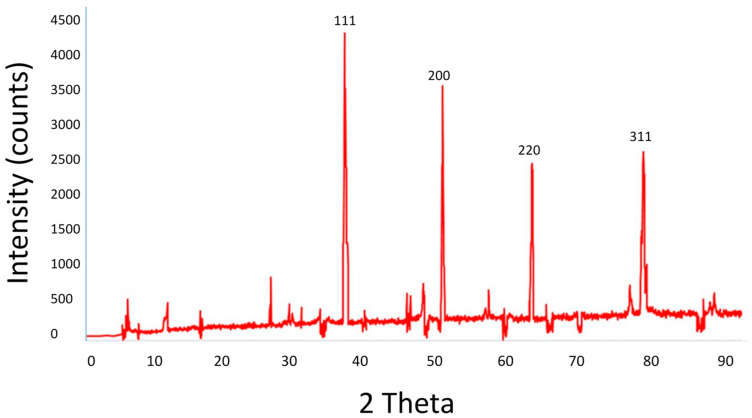
XRD patterns of biosynthesized *Bauhinia. tomentosa* Linn flower-powder extract-derived AgNPs. The XRD pattern displays diffraction peaks at 2θ = 37°, 49°, 63°, and 76° (corresponding to the planes of silver 111, 200, 220, 311), respectively. The XRD data and pattern confirmed the crystalline structure of biosynthesized AgNPs. No significant peaks corresponding to other crystalline phase impurities were detected.

The XRD method was used to determine the crystalline phase of the biosynthesized *B. tomentosa* Linn flower-powder extract-derived AgNPs. The XRD pattern includes diffraction peaks at 2θ = 37°, 49°, 63°, and 76°, corresponding to the planes of silver (111, 200, 220, 311), respectively (Figure 4). The XRD data and pattern confirmed the crystalline structure of the biosynthesized AgNPs. No significant peaks corresponding to other crystalline phase impurities were detected. All peaks in the XRD pattern can be assumed to correspond with the structure of silver.

### 3.6. Scanning Electron Microscopic Analysis Biosynthesized AgNPs

**Figure 5:**
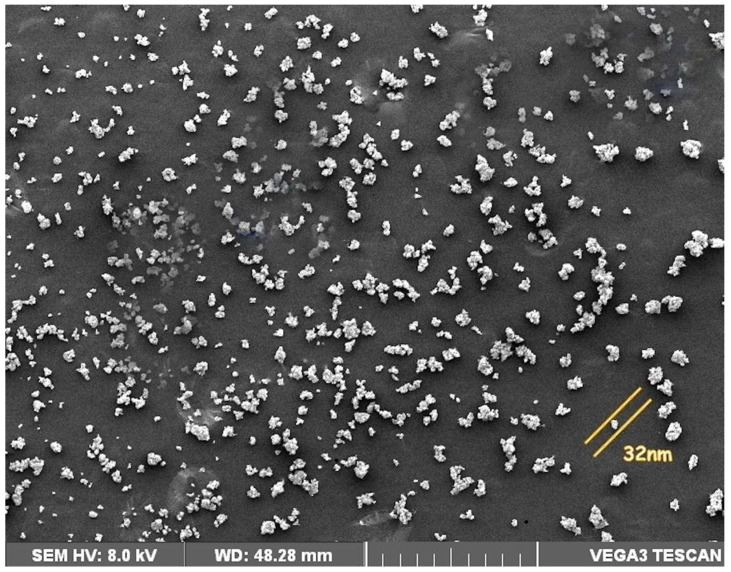
SEM analysis of biosynthesized *Bauhinia tomentosa* Linn flower-powder extract-derived AgNPs.

An SEM analysis revealed uniformly distributed AgNPs on the surfaces of the nanoparticles. An SEM image of silver nanoparticles synthesized using *B. tomentosa* Linn flower extract shows spherical and relatively uniform shapes with a diameter near 32 nm (Figure 5).

### 3.7. Antibacterial Activity of Biosynthesized AgNPs

**Figure 6:**
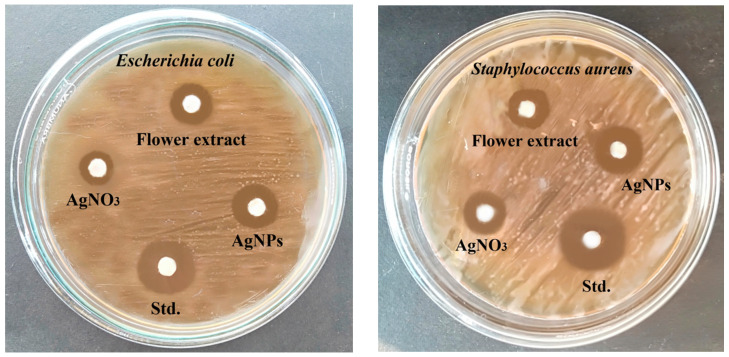
Qualitative antibacterial (*Escherichia coli* (**left**) and *Staphylococcus aureus* (**right**)) activity of biosynthesized *B. tomentosa* Linn flower-powder extract-derived AgNPs. The discs were dipped with the following four different components (30 μL): (i) biosynthesized AgNPs, (ii) *Bauhinia tomentosa* Linn flower powder extract, (iii) AgNO_3_ solution, and (iv) standard antibiotic solutions (chloramphenicol, 30 µg/mL).

**Figure 7:**
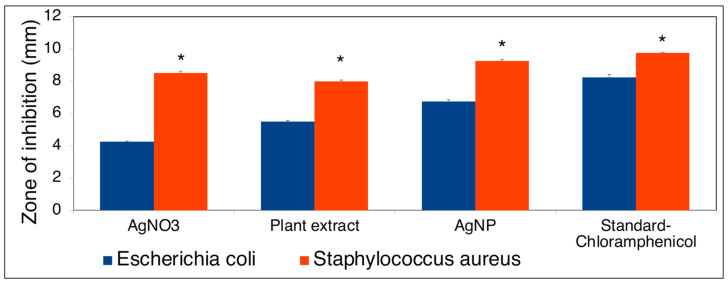
Quantitative antibacterial (*Escherichia coli* (blue) and *Staphylococcus aureus* (red)) activity (measurements of zone inhibition activity) of biosynthesized *Bauhinia tomentosa* Linn flower-powder extract-derived AgNPs (as shown in Figure 6). Data are presented as mean ± standard deviation of four independent experiments (* *p* < 0.01 [*E. coli* compared with *S. aureus*]). AgNP has the same efficacy as chloramphenicol.

The antibacterial activity of the biosynthesized AgNPs was determined using disk diffusion. The antibacterial activity of the biosynthesized AgNPs tested against Gram-negative (*E. coli*) and Gram-positive (*S. aureus*) bacterial pathogens showed a larger zone of formation against *S. aureus* (9.25 mm ± 0.956 mm) compared with that of *E. coli* (6.75 mm ± 0.957 mm) (Figure 6 and Figure 7).

### 3.8. Antifungal Activity of Biosynthesized AgNPs

**Figure 8:**
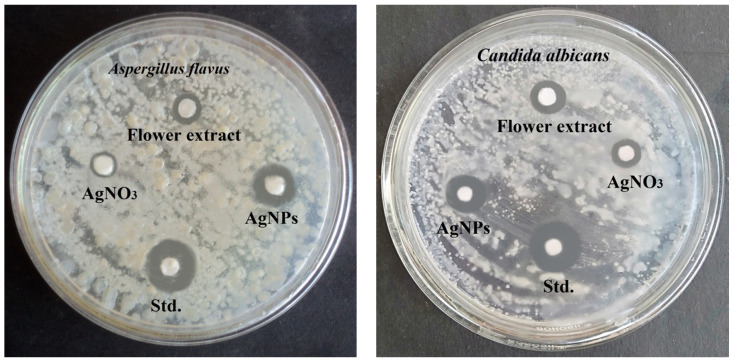
Qualitative antifungal activity of of biosynthesized *Bauhinia tomentosa* Linn flower-powder extract-derived AgNPs against fungal strains *Aspergillus flavus* (**left**) and *Candida albicans* (**right**). The following four different components (30 μL) were applied on separate Whatman No. 1 filter paper discs 6 mm in diameter: (i) biosynthesized AgNPs, (ii) *Bauhinia tomentosa* Linn flower powder extract, (iii) AgNO_3_ solution, and (iv) standard antifungal solution (fluconazole, 30 µg/mL), which were allowed to dry before being placed on a potato dextrose agar medium carrying the fungal strains and incubated for 48 h. The diameters of the zones were measured in centimeters.

**Figure 9:**
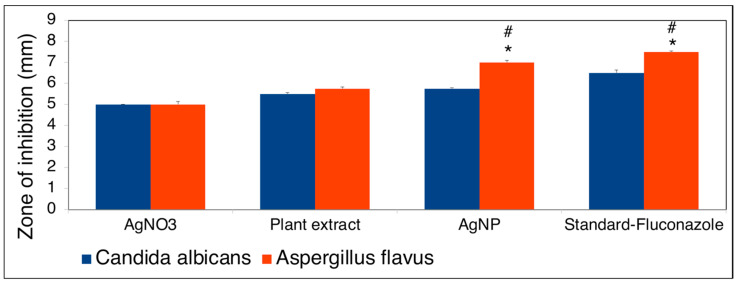
Quantitative antifungal strains (*Aspergillus flavus* (red) and *Candida albicans* (blue)) activity (measurements of zone inhibition activity) of biosynthesized *Bauhinia tomentosa* Linn flower-powder extract-derived AgNPs (as shown in Figure 8). Data are presented as mean ± standard devation of four independent experiments (* *p* < 0.01 [*A. flavus* compared with *C. albicans*], # *p* < 0.1 [compared with AgNO_3_ and plant extract, respectively]).

The antifungal activity of biosynthesized *B. tomentosa* Linn flower-powder extract-derived AgNPs was determined by disk diffusion against the fungal strains *A. flavus* and *C. albicans*. Fluconazole was used as a standard antifungal agent. The AgNPs achieved superior inhibition against *A. flavus* (zone of inhibition: 7 ± 0.812 mm) compared with *C. albicans* (zone of inhibition 5.75 ± 0.447 mm) (Figure 8 and Figure 9).

### 3.9. Antioxidant Activity of Biosynthesized AgNPs

**Figure 10:**
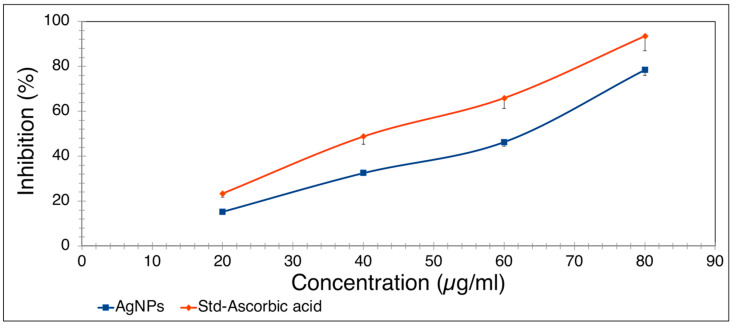
Dose-dependent antioxidant activity of biosynthesized *Bauhinia tomentosa* Linn flower-powder extract-derived AgNPs. Ascorbic acid served as a positive control. The indicated mean values are from two independent experiments performed in triplicate (maximum mean deviation ± 5%).

The radical scavenging activity of biosynthesized *B. tomentosa* Linn flower-powder extract-derived AgNPs was quantified spectrophotometrically by changing the DPPH color from brown to yellow. Inhibition of DPPH radical scavenging activity was found to be dose-dependent with half-maximal inhibitory concentration (IC_50_) values of 56.77 μg/mL and 43.03 μg/mL for AgNPs and ascorbic acid (control), respectively (Figure 10).

### 3.10. Molecular Docking of Biosynthesized AgNPs

**Figure 11:**
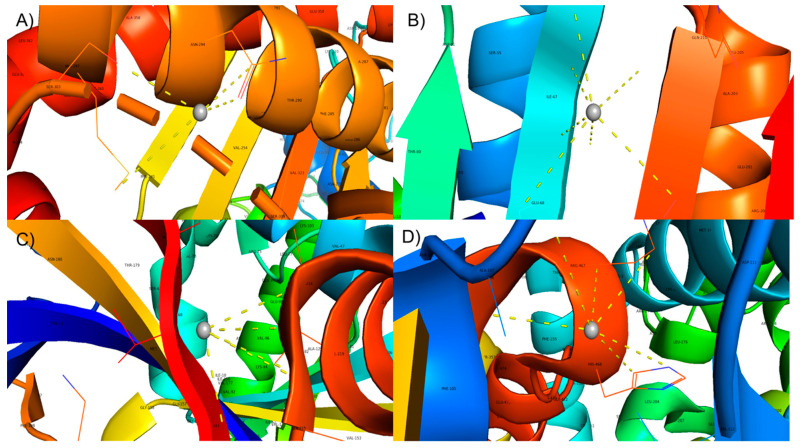
Molecular docking of AgNPs determines the binding ability of silver with various bacterial and fungal proteins. A 3D structure modeling of the interaction of silver (gray ball in the center of each subdisplay (**A**–**D**)) with bacterial species: (**A**) *Staphylococcus aureus*, (**B**) *Escherichia coli*; and with fungal species: (**C**) *Candida albicans*, (**D**) *Aspergillus flavus*. AgNP interactions with microbes were achieved by hydrophobic contact. PDB IDs used included 3G7B (DNA gyrase, *Staphylococcus aureus*), 4WUB (DNA gyrase, *Escherichia coli*), 5TZI (cytochrome P450, *Candida albicans*), and 6DRS (dihydrofolate reductase, *Aspergillus flavus*).

The antimicrobial mechanisms of AgNPs against bacterial or fungal pathogens remain unclear. AgNPs can directly attack and disrupt or penetrate cell walls to induce intracellular redox reactions mediating cytotoxicity. Moreover, AgNPs can interact with pivotal microbial proteins to inhibit their activities and cause cell death [78,79,80,81]. Accordingly, we selected representative proteins for each species to study the possible three-dimensional (3D) interaction of AgNPs with bacterial DNA gyrase [82,83], fungal CYP51 (cytochrome P450 monooxygenase (CYP) superfamily) [51,84] and fungal dihydrofolate reductase [85]. To predict the biological interactions of the biosynthesized *B. tomentosa* Linn flower-powder extract-derived AgNPs with these possible microbial target proteins, we performed molecular docking analysis using a Patch dock server for the 3D structures of PDB proteins 3G7B (DNA gyrase, *S. aureus*), 4WUB (DNA gyrase, *E. coli*), 5TZI (cytochrome P450, *C. albicans*), and 6DRS (dihydrofolate reductase, *A. flavus*). Silver nanoparticle bound microbe structures (DNA gyrase, cytochrome P450, and dihydrofolate reductase) were visualized for interaction by PyMOL (Version 2.3.0, PyMol Molecular Graphics system, Schrödinger, LLC, New York, NY, USA). By the molecular rendering approach, interaction of AgNPs with amino acid (AAs) in the target protein structures was identified. The AA residues interacted with silver through hydrophobic contact (Figure 11). 

## 4. Discussion

We biosynthesized AgNPs using the natural extract of *B. tomentosa* Linn. We then applied various biophysical and biochemical methods to characterize the potential biomedical applications of the AgNPs [8,9,10,12,13,14,15,16,86] and validated their antimicrobial and antioxidant properties [87,88]. We also evaluated a possible mechanism of action via molecular docking analysis.

We applied multiple biophysical and biochemical methods to characterize our biosynthesized AgNPs. A UV-vis spectroscopic analysis showed a characteristic absorbance peak shift from 400 nm to 420 nm during the formation of biosynthesized *B. tomentosa* Linn flower-powder extract-derived AgNPs (Figure 1), which can be attributed to the formation of larger particles [57,89,90]. An EDX analysis helped demonstrate the elemental composition of the biosynthesized *B. tomentosa* Linn flower-powder extract-derived AgNPs (Figure 3, Table 2). The dense peak corresponding with silver strongly confirmed the reduction of AgNO_3_ and the formation of AgNPs. An EDX analysis also proved that the required phase of silver was present in the biosynthesized AgNPs [27,61,63,76,91]. The crystalline nature of the biosynthesized AgNPs was confirmed in the form of XRD diffraction peaks at 2θ = 37°, 49°, 63°, and 76° (corresponding to the planes of silver 111, 200, 220, 311), respectively (Figure 4), which are typical XRD values of biosynthesized AgNPs [65,76,92,93,94]. Additionally, FTIR spectroscopy confirmed the various functional (amine, alkyl, ether, and aliphatic) groups and chemical bonding of biosynthesized AgNPs, while SEM analysis revealed the surface morphology and size of the AgNPs, which assumed spherical, uniform shapes (Figure 5) [11,61,62].

To determine possible biomedical applications of the biosynthesized *B. tomentosa* Linn flower-powder extract-derived AgNPs we examined their potential antimicrobial activity. The biosynthesized AgNPs exhibited efficient anti-Gram-negative and anti-Gram-positive bacterial activity, with higher efficiency against Gram-positive bacterial pathogens (Figure 6 and Figure 7). Moreover, the biosynthesized AgNPs exhibited significant antifungal activity, as determined by the disk diffusion method, against *A. flavus* and *C. albicans*, respectively (Figure 8 and Figure 9). Recent data point to the possible redox-potential of *B. tomentosa* Linn-derived AgNPs and their possible uses as antimicrobial agents [67]. The antimicrobial activity of our biosynthesized AgNPs may be mediated by a redox reaction, which was confirmed by the reduction and radical scavenging potential of silver in green biosynthesized AgNPs (against DPPH). The lowest concentration of the biosynthesized *B. tomentosa* Linn flower-powder extract-derived AgNPs was 20 μg/mL, with an effectivity of 15.30 ± 0.40% and an IC_50_ of 56.77 (Figure 10), which was superior and in the range of previously described AgNPs using other green sources [63,95,96]. Therefore, our results presented here indicate that our biogenic AgNPs are superior to other biosynthesized AgNPs in terms of higher in vitro antioxidant [34,47,95] and higher in vitro antimicrobial efficacy (Table 3) [34,45,47,56,57,58,61,62,63,65,69,70,96].

Finally, we used molecular modeling and docking analyses to investigate the antibacterial and antifungal mode of action of the biosynthesized AgNPs. We observed AgNP-mediated cytotoxicity and identified the AA residues SER-303, ASN-294 (DNA gyrase from *Escherichia coli*), ILE-67, THR-212, GLN-210 (DNA gyrase from *S. aureus*), ALA-107, PHE-105 (cytochrome P450 from *C. albicans*), and VAL-214, ALA-216 (dihydrofolate reductase from *A. flavus*) as possible participants in hydrophobic interactions with validated silver in the biosynthesized AgNPs, which are potentially responsible for the antibacterial and antifungal redox reactions mediating microbial cytotoxicity. We inferred from molecular modeling and docking studies that the biosynthesized AgNPs can effectively bind to microbes and act as antimicrobial agents (Figure 11) [66,70,99].

**Figure 12:**
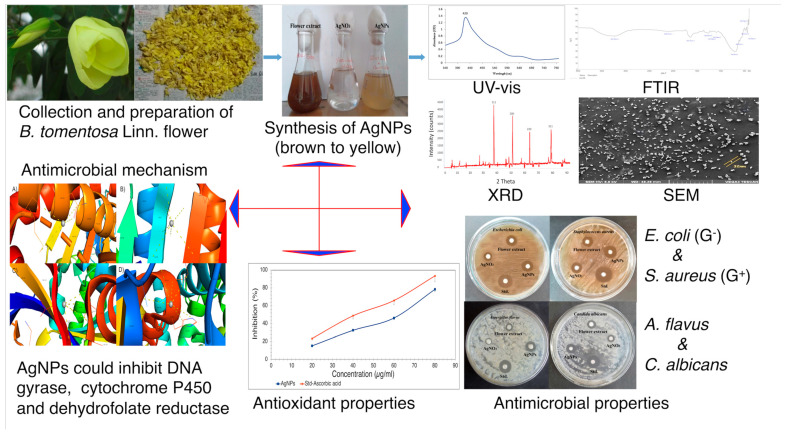
Overview of the study of *B. tomentosa* Linn flower extract-derived biogenic AgNPs. Biosynthesized AgNPs (change in the color (brown to yellow) of the solution over time when aqueous plant extract was added to a AgNO_3_ solution). Biophysical characterization of biosynthesized AgNPs by UV-vis, FTIR, XRD, and SEM confirmed the nature of AgNPs. Biochemical and cellular analyses confirmed the antioxidant (dose-dependent DPPH radical scavenging activity) and antimicrobial (antibacterial (Gram-positive (G^+^) and Gram-negative (G^−^)) and antifungal) properties of the biogenic AgNPs. Molecular modelling and docking studies indicated the possible antimicrobial activity mechanism of the biogenic AgNPs: inhibition of key enzymes such as DNA gyrase, cytochrome P450, and dihydrofolate reductase.

Thus, the use of *B. tomentosa* Linn extracts for the synthesis of biomedically important AgNPs therefore has several advantages, since the environmentally friendly synthesis provides stable and highly effective AgNPs with a highly effective redox potential for highly effective antimicrobial activity and possible biomedical applications (Figure 12) [7,34,41,43,44,45,46,47].

## 5. Conclusions

Silver nanoparticles from different natural sources are useful industrial and medicinal tools. *B. tomentosa* Linn flower-powder extract-derived AgNPs were characterized through UV-vis spectrophotometry, FTIR, XRD, and EDX. We observed the reduction of Ag^+^ to Ag^0^ with an accompanied UV-vis spectral peak shift from 400 nm to 420 nm over 4 h. The FTIR analysis revealed the functional (amine, alkyl, ether, and aliphatic) groups of AgNPs, while XRD analysis showed that the biosynthesized AgNPs had a crystalline structure. Results of SEM analysis revealed the AgNPs were spheres approximately 32 nm in diameter. The results of EDX examination confirmed the presence of Ag^0^ in biosynthesized AgNPs with reducing antioxidant properties validated by DPPH assays. Biologically synthesized AgNPs exhibited antibacterial activity against *E. coli* (Gram-negative) and *S. aureus* (Gram-positive) as well as antifungal activity against *C. albicans* and *A. flavus*. A possible mode of reducing antibacterial and antifungal activities was studied by molecular docking analysis, which indicated that the biosynthesized *B. tomentosa* Linn flower-powder extract-derived AgNPs may be able to inhibit key enzymes, such as bacterial DNA gyrase and fungal cytochrome P450 (*C. albicans*) and dihydrofolate reductase (*A. flavus*). This study may pave the way for the development of new and potentially antimicrobial compounds based on biosynthesized *B. tomentosa* Linn flower-powder extract-derived AgNPs (Figure 12).

## Figures and Tables

**Table 1:** Phytochemicals present in aqueous and alcoholic extracts of *B. tomentosa* Linn.

S. No.	Test	Aqueous Extract	Alcohol Extract
1	Alkaloids	+	+
2	Antroquinone	++	+
3	Coumarins	++	+
4	Flavonoids	++	+
5	Glycoside	+	+
6	Polyphenol	++	+
7	Saponin	++	+
8	Steroids	++	+
9	Tannin	+	−
10	Terpenoids	+	+
11	Triterpenoids	+	+

Note: “+” = present, “++” = strongly present, “−” = absent.

**Table 2:** EDX elemental composition of biosynthesized *B. tomentosa* Linn flower-powder extract-derived AgNPs.

Elements	Atomic Number (Periodic Table of Elements)	Shells	Weight %	Atomic %
**Ag**	47	L-series	75.86	59.08
**Cl**	17	K-series	25.14	41.92
**Total**			100	100

**Table 3:** Comparative antimicrobial efficacy of biosynthesized *B. tomentosa* Linn flower-powder extract-derived^$^ AgNPs.

	Antioxidant Efficacy, AgNPs ^$^; IC_50_ (AgNPs & Ascorbic Acid (Control), Respectively) [μg/mL]	Antioxidant Efficacy, Other AgNPs; IC_50_ (AgNPs & Ascorbic Acid (Control), Respectively) [μg/mL]	Antimicrobial (Antibacterial) Efficacy, AgNPs ^$^; Zone Inhibition [mm]	Antimicrobial (Antibacterial) Efficacy, Other AgNPs; Zone Inhibition [mm]	Antimicrobial (Antifungal) Efficacy, AgNPs ^$^; Zone Inhibition [mm]	Antimicrobial (Antifungal) Efficacy, Other AgNPs; Zone Inhibition [mm]	References
**1**	56.77 & 43.03	50.37 & 44.10					[47,63]
**2**		46.25 & 41.86					[95]
**3**			6.75 (*E. coli*) (30 μL)	11.4 (*E. coli*) (50 μL)			[57,67]
**4**			9.25 (*S. aureus*) (30 μL)	12.7 (*S. aureus*) (50 μL)			[56,67]
**5**					5.75 (*C. albicans*) (30 μL)	10.7 (*C. albicans*) (50 μL)	[39,97,98]
**6**					7 (*A. Flavus*) (30 μL)	20 (*A. Flavus*) (50 μL)	[97,98]

## Data Availability

Data is contained within the article.
